# Stable atmospheric conditions underlie a steady pace of nocturnal bird migration in the tropics

**DOI:** 10.1098/rspb.2024.2609

**Published:** 2025-06-11

**Authors:** Jacob R. Drucker, Benjamin M. Van Doren, Nicholas J. Bayly, Wilmer Ramirez, Alfonso Ladino Rincon, John M. Bates, Adriaan M. Dokter

**Affiliations:** ^1^ Committee on Evolutionary Biology, The University of Chicago, Chicago, IL 60637, USA; ^2^ Negaunee Integrative Research Center, Field Museum of Natural History, Chicago, IL 60606, USA; ^3^ Department of Natural Resources and Environmental Sciences, University of Illinois Urbana-Champaign, Urbana, IL 61801, USA; ^4^ SELVA: Research for Conservation in the Neotropics, Bogotá, Bogotá D.C. 111311, Colombia; ^5^ Happy Life Guaviare, San José del Guaviare, Guaviare 950001, Colombia; ^6^ Department of Climate, Meteorology, and Atmospheric Science, University of Illinois at Urbana-Champaign, Urbana, IL 61801, USA; ^7^ Cornell Lab of Ornithology, Cornell University, Ithaca, NY 14850, USA

**Keywords:** aerocology, tropical ecology, bird migration, weather surveillance radar, acoustic monitoring

## Abstract

Seasonal migrations between temperate and tropical latitudes have evolved repeatedly across the tree of life, but we know little about how tropical environments have shaped this life history strategy. In this first aeroecological radar study in South America, we tested whether tropical atmospheric stability leads to a more continuous pace of nocturnal bird migration across a season than the episodic pulses of migration in temperate regions. We estimated and compared the volume, flight directions and heights of birds detected by weather radars and acoustic sensors in three regions of Colombia across four years and assessed the effects of atmospheric conditions. We found that stable tropical winds have distinct effects on migration timing depending on geographic context. Within the Andes mountains, supportive winds explain the altitudes at which birds fly, but not the volume of birds aloft on a given night, resulting in lower nightly variation in migration intensity. However, lapses in prevailing headwinds east of the Andes drove higher variation in migration intensity similar to temperate latitudes. With a reduced relationship between forecastable weather and the magnitude of nightly migration, predicting high volume migration events to target for conservation initiatives may be more challenging in tropical climates.

## 1. Introduction

The seasonal migrations of birds and other flying organisms between temperate and tropical latitudes are structured in space and time by atmospheric circulation patterns that can help or hinder flight, as well as geographic barriers that may be associated with physiological challenges and mortality risk [1–3]. The role of these factors in defining migration dynamics through North America and Eurasia are increasingly well understood, informing novel conservation strategies and deepening scientific understanding about the ecology and evolution of animal migration [3–6]. However, these insights remain biased towards temperate regions, leaving a critical knowledge gap about migration in tropical regions where many species that breed at temperate latitudes spend the majority of their annual cycle [7]. Further study in tropical climates and landscapes is needed to synthesize a holistic view of how bird migration functions and may evolve under different environmental regimes, particularly given recent population declines and rapid global change [8,9].

A fundamental difference between tropical and temperate climates is the scale of regional weather systems that determine the nightly variability in the magnitude of migration. At temperate latitudes, extratropical cyclones drive extreme variation in wind patterns that operate at large spatial and short temporal scales, both aiding and impeding bird movement across continents and seas and shaping extant migratory flyways [2]. Frontal weather systems at temperate latitudes create favourable flight conditions for one region while inhibiting flight in another, yielding episodic pulses of migration in different parts of the continent across a time span of several days [10]. This relative scarcity of optimal flight conditions during a migration season means that a large percentage of passage occurs over relatively few nights in a season [11]. In contrast, local climates in tropical regions, like northern South America, are more stable in time but heterogeneous in space, with unclear effects on the temporal and spatial distribution of bird migration. Because the Intertropical Convergence Zone suppresses large wind fields and the influence of the trade winds, moisture sources interacting with topography promote weather patterns that may vary more within a day than across a year, often at a fine geographic scale [12,13]. As a result, supportive or opposing winds for migration may not occur at a scale that translates into episodic pulses of bird migration over mainland South America or regional differences in the number of birds aloft [2,10,14]. For these reasons, low variation in the magnitude of nightly migration may be expected in tropical regions, translating into a steady pace of migration across a season. Alternatively, it is possible that seasonal migration still occurs in episodic pulses in the absence of frontal weather systems. Coordinated departures in the fall from North America and Caribbean Islands based on local weather may translate into highly variable volumes of nightly migration when birds reach South America [15]. In spring, the same pattern may also be driven by timed departures from overwintering destinations based on regional precipitation trends [16].

The altitudes where birds can find profitable winds may also affect spatial variation in migration by determining how high they fly [17–19]. Air rotation cells at mid-latitudes drive prevailing winds that blow roughly longitudinally but are subject to interruption from extratropical cyclones, while between the equator and 30° north and south, the north-east and south-east trade winds and other low-level jets blow consistently in the same direction but at varying altitudes [12]. Birds experiencing temperate conditions have been shown to prefer lower altitudes, often flying only as high as necessary to avoid opposing winds and forming vertical layering in cases where supportive winds are only found higher up [17–19]. But the consistent vertical layering of opposing and supportive wind in the trade-wind zone (i.e. between 15° and 30°) means that birds migrating through this region more often stratify altitudinally, flying higher or lower depending on whether the jet is beneficial during their north- or south-ward migrations, tracking supportive winds to altitudes as high as 9000 m.a.s.l [20,21,22]. If and how the influence of the trade winds on migration dissipates closer to the Intertropical Convergence Zone and the equator remains undocumented. Other regional low-level jets drive local climates in the tropics, but no study has examined their effects on animal migration [12,23].

Patterns of local migration may also be shaped by physical barriers that act as navigational cues, leading lines or funnels, such as large water bodies and mountain ranges [15,24–26]. However, the interaction between climate, topography and migration remains poorly studied across latitudinal contexts. For example, conditions with precipitation and low visibility that ground active migrants are typically associated with individual weather events at temperate latitudes but are a regular fixture of the climate in tropical mountains [10]. Corpses of migrants found on glaciers in the Andes mountains following periods of low visibility are a testament to the compounded challenge of crossing giant mountains in adverse conditions [27]. Additionally, geographic context, including longitude, the size and shape of a land mass and proximity to critical breeding, non-breeding or stopover habitat may affect the spatio-temporal patterns of migration across the landscape [28]. In the Americas, birds typically have significantly more land mass available in their temperate breeding distributions than in their tropical non-breeding distributions, leading to higher densities of individual species in Central and South America [29,30]. However, latitude shapes the weather that interacts with key geographic features like mountains to create different conditions for migratory birds throughout their journeys [30]. That is, tropical mountain ranges may present different challenges to migratory birds than temperate mountains.

We characterized the spatiotemporal dynamics of bird migration across a tropical landscape by measuring the passage of migratory landbirds that breed in North America and spend the boreal winter in South America (hereafter ‘boreal migrants’, contrasting with ‘austral migrants’ breeding in South America) over three biogeographic regions of Colombia using weather surveillance radars and acoustic monitoring. Weather radars have revolutionized the scale at which migration can be quantified but have rarely been used for this purpose in the tropics, and never in South America [14,31–36]. Similarly, increasingly proficient sound classification models have expedited the utility of autonomous acoustic monitoring for birds migrating at night but have not been leveraged at scale in the tropics [37]. As of 2018, the Colombian government has implemented a network of weather radars that offer a novel opportunity to measure how birds migrate through this country that serves as a critical migratory pathway into and out of South America. We integrated measures of bird densities and flight directions from weather radars and call rates from recording stations to quantify spatial variation in the volume, direction, height, timing and species composition of nocturnal migration relative to wind support and local orographic features. We subsequently test the hypothesis that reduced variability in supportive wind drives a steady, rather than episodic, pace of bird migration across Colombia based on the predictions that (i) nightly variation in the magnitude of migration is low relative to a representative temperate location, (ii) migration is spread relatively evenly across a season instead of concentrated within a small percentage of nights, and that (iii) nightly bird density responds weakly to supportive winds, because winds near the equator are typically weak and they vary at finer spatial scales [13,23]. We additionally explore the influence of biogeographic regions and topographic features such as inter-Andean valleys on the magnitude of local migration in Colombia.

## 2. Methods

### (a) Compiling radar data

**Figure 1.**
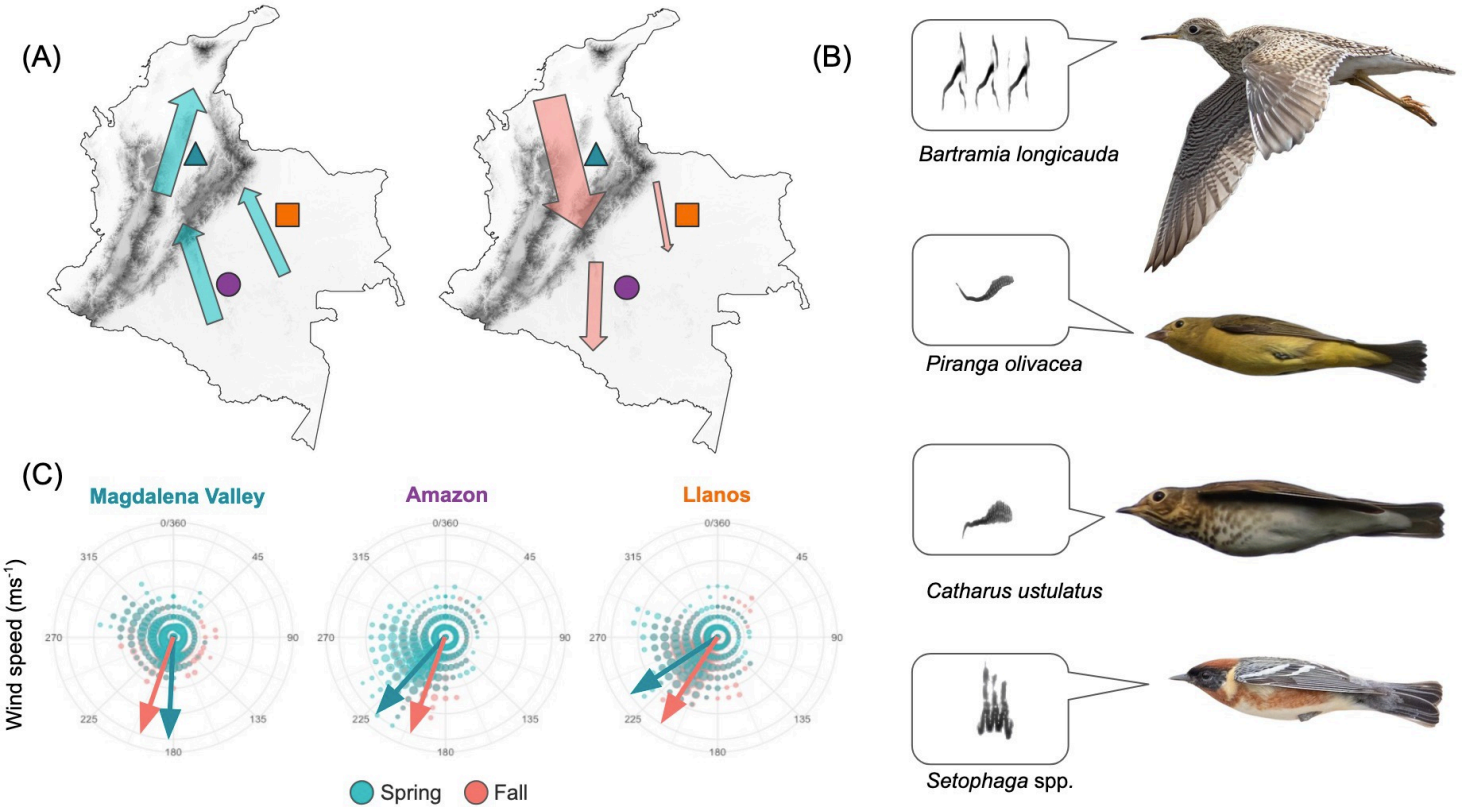
Overview of geography, species composition and wind regime in the study region. (A) Map of Colombia showing the magnitudes (proportional to areas of arrows) and directions of bird migration over each focal radar station during the boreal spring (blue) and fall (red) migrations. Bird densities and directions are detailed in electronic supplementary material, figures S9 and S10 and table S2.. Focal radar stations are coloured according to biogeographic region: teal for the Magdalena Valley (BAR radar), orange forthe Llanos grasslands (CAR radar) and violet for the Amazon (GUA radar). Grey shading illustrates topographic relief, from sea level to >4000 m.a.s.l. (B) Spectrograms and images of representative species that migrate through Colombia en route to non-breeding grounds, based on acoustic monitoring. Bird photos courtesy of J. Drucker, D. Gochfeld, M. O’Brien and I. Davies. (C) Rose plots showing distribution of wind speed and direction available to birds over each radar station during boreal spring and fall. Distance from the plot centre indicates speed, with values of 0 m s^−1^ at the centre of the circle and values of 20 m s^−1^ at the edge of the circle. Arrows depict mean seasonal directions.

We compiled raw weather surveillance radar data from three Vaisala wrm 200 C-band radars operated and maintained by Colombia’s Institute for Hydrological, Meteorological and Environmental Studies (IDEAM), amounting to 370 782 polar volume scans on 1465 nights between September 2018 and December 2022 across three stations. We estimated vertical profiles of bird density, speed and direction using the algorithm vol2bird [38] available in the R package bioRad [39]. Polar volume data were obtained in IRIS RAW format from the s3-radaresideam public S3 bucket, managed by the IDEAM and distributed under the Amazon Web Services registry of open data [40]. From 2018 to June 2021, we only used polar volumes labelled with an IRIS RAW task equal to ‘WIND’. In June 2021, the WIND task was replaced with PREC_A, PREC_B and PREC_C tasks, each file containing a subset of elevation scans. We merged these partial volumes into a single polar volume containing all available elevation scans before calculating a profile. Vertical profiles were calculated using 100 m altitude bins up to 5000 m above sea level, using data in the 5 and 35 km range only. We used unfiltered reflectivity (TH) instead of Doppler-filtered reflectivity (DBZH) to calculate profiles, because we found that Doppler filters were overly aggressive in removing weak biological echoes [41]. Only close-range data were used to maintain a sufficiently narrow beam for sampling altitudinal distributions. Radars were located in three biogeographically distinct regions (figure 1A, electronic supplementary material, figure S1): (i) the Magdalena River Valley (hereafter ‘Magdalena Valley’) nestled between the Central and Eastern Andean Cordilleras (Barrancabermeja (BAR) radar, *n* = 1 71 986 volumes [latitude,longitude]=[6.9325°−73.7625°], height = 80 m.a.s.l.), (ii) the Llanos or Orinoco plains, a tropical savanna (Carimagua (CAR) radar, *n* = 80 172 scans [4.5636°−71.3325°], height = 181 m.a.s.l.), and (iii) the northern edge of the Amazon basin (San Jose del Guaviare (GUA) radar, *n* = 1 18 624 scans [2.5336°−72.6283°], height = 218 m.a.s.l.). These three radars are a subset of the stations currently operated in Colombia [40] and we selected them based on data completeness and their location in lowlands that allow for broad spatial coverage with minimal beam interference from nearby topography. To provide a basis of comparison with temperate latitudes, we also calculated vertical profile time series of radar data from three mid-latitude stations from the NEXRAD weather surveillance radar network in the United States from 2018, 2019 and 2021, concurrent with our primary Colombian dataset: KLNX in central Nebraska [41.95778°−100.5764°, 949 m.a.s.l], KLOT in northern Illinois [41.60472°−88.08472°, 232 m.a.s.l.] and KBGM in central New York [42.19972°−75.98472°, 519 m.a.s.l]. These stations are evenly spaced longitudinally across Eastern North America, capturing longitudinal variation in the migration flow of this avifauna.

We paired radar data with the North American Regional Reanalysis (NARR) to obtain measures of atmospheric conditions at all altitudes scanned by the radar [42]. Atmospheric data were available every 3 h at 15 pressure levels between 1000 and 600 hPa and were matched to the altitude layers and time stamp of the vertical profile by linear interpolation in time followed by altitude, using data from the nearest atmospheric grid point to the radar. Because Colombia lies near the geographic limits of this weather product, we verified its accuracy by downloading weather balloon data from the same geographic regions as the radars (SKBG balloon in Bucaramanga, approx. 75 km from BAR radar in the Magdalena Valley and SKTQ balloon in Caquetá, approx. 350 km from the GUA radar in Amazonia, Wyoming Weather Web accessed November 2021) and compared readings of wind speed and direction between the balloons and the NARR on dates with concurrent sampling during 2018, 2019 and 2020. We were unable to use weather balloon data directly in this study because balloon readings were taken infrequently, but they served as a useful validation for NARR data. We found moderately low differences in wind speeds measured by these two metrics that are lower than the typical self-propelled speeds of birds (mean absolute difference 5.5 m s^−1^ between model and balloon in Magdalena Valley, 6.8 m s^−1^ between model and balloon in Amazonia, electronic supplementary material, figure S2). We therefore concluded that NARR data were sufficiently accurate for the purposes of this study.

### (b) Processing polar volumes into vertical profiles of birds

Biological targets detected by weather radars in the tropics potentially include large numbers of aerial insects in addition to birds. We partitioned bird and insect signal contributions using prior knowledge on the expected airspeed for these two taxa [33,43]. We employed a model that assumes insects move approximately at the same speed and in the same direction as the wind, and that birds are self-propelled, faster-flying targets moving independently of the wind [14]. Assuming fixed airspeeds for birds and insects during prolonged, active flight allows us to calculate the proportion of birds at each altitude and time interval, which we multiplied with reflectivity to estimate the total reflectivity owing to birds, or ‘bird-reflectivity’ (electronic supplementary material, figure S3). This model also derives the north–south ($v_{bird}$) and east–west ($u_{bird}$) groundspeed components of the birds in the radar volume, which we used to derive bird headings *φ *= 90 − (180/π) × arctan2($u_{bird},v_{bird}$) and groundspeed direction $dd_{bird}$ = 90 − (180/π) × arctan2$\left(u_{bird}+u_{wind},v_{bird}+v_{wind}\right)$, with arctan2, the four-quadrant inverse tangent (electronic supplementary material, table S2). We assumed a representative fixed insect airspeed of 1 m s^−1^ and bird airspeed of 8 m s^−1^ when calculating bird proportions based on empirically measured self-propelled speeds of insects and passerine birds [33,43,44]. Slightly increasing or decreasing assumed fixed airspeeds between 0.5 and 3.0 m s^−1^ for insects and 8 and 12 m s^−1^ for birds did not qualitatively change our results [14]. We estimated tailwind assistance using the EQ_airspeed_ model of flow assistance in [45], inputting the bird-reflectivity-weighted mean ground speed direction (*dd*_bird_) for each station and season as the ‘preferred migratory direction’ variable.

Given our focus on Neotropical migrant landbirds, we further cleaned our dataset by removing cases with headings inconsistent with birds’ seasonally preferred migration direction. That is, we removed altitude layer data with southbound ground speed directions (i.e. >90° and <270°) in the boreal spring, when birds are migrating north, and we removed cases with northbound headings (i.e. <90° and >270°) in the boreal fall, when birds are migrating south. While this approach may overlook patterns of reorientation and other potentially significant regional bird movements (e.g. movement to and from roosting locations), we believe it most accurately captures the directed, broad-front movements of interest in this study and is an added safeguard against contamination by non-avian taxa. We also excluded all data outside the migratory periods in Colombia and Illinois, defined here as February 20 to June 1 and August 1 to November 25 for boreal spring and fall, respectively. We excluded data between sunrise and sunset to maintain a focus on nocturnal migration.

### (c) Analysis of vertical profile time series

We quantified migration phenology across a season by first integrating vertical profile time series across all altitudes for each polar volume with the integrate_profile() function in the R package ‘bioRad’ [39]. Using a radar cross section of 11 cm^2^, this yielded measures of vertically integrated bird density (*VID*, unit birds km^−2^), hereafter ‘bird density’ [38], migration traffic rate or ‘*MTR*’, and total cumulative ‘migration traffic’, or ‘*MT*’. We were unable to estimate migration traffic in the Llanos owing to a lack of continuous nightly data throughout an entire season when the radar was not operational.

To assess nightly variability in the magnitude of migration, we calculated the mean and coefficient of variation of migration traffic rate for stations with more than two weeks of continuous data and total migration traffic for seasons with at least 75% complete time series (electronic supplementary material, table S1) based on a total of five seasons in the Magdalena Valley (Spring 2019, Fall 2020, 2018, 2019, 2021), five seasons in Amazonia (Spring 2019, 2020, Fall 2018, 2019, 2022) and six seasons in Illinois, Nebraska and New York, USA for a basis of comparison with temperate latitudes Spring and Fall of 2018, 2019 and 2021). To ensure that observed differences in *MTR* variability between years were not affected by sample sizes, we bootstrapped these mean and coefficient of variation values with 10 000 replicates to generate 95% confidence intervals and compared the bootstrap replicates between sites and seasons using an ANOVA with *post-hoc* Tukey tests. We further quantified nightly variation in migration traffic by identifying the percentage of nights across a season required to capture 50%, 75% and 90% of total seasonal migratory passage. If the pace of migration is more episodic, fewer nights are needed to capture half of seasonal migration, and if migration is more steady, high volume migration should be more spread out across a larger percentage of nights [11]. Seasonal migration traffic totals were compared for complete or near-complete time series (i.e. with >3000 volumes). We repeated this analysis for the ‘core’ of each of these seasons, focusing on the number of days required to capture 20–80% and 10–90% of total traffic.

To directly compare the magnitude and altitude of migration between sites and seasons, we calculated the mean values of bird density and flight height for each night with the three Colombian radars operating simultaneously.

To interpret the effects of weather on bird densities, we thinned the full dataset to one scan per night for each station at or closest to 3 h after sunset, when migration typically reaches peak densities [46]. This approach eliminates pseudoreplication within nights and differences in sample sizes between sites. Furthermore, using scans well after sunset avoids contamination from concentrations of bats emerging from roost sites at dusk. We only modelled data for years with identical radar scan settings (i.e. IRIS task ‘WIND’ only), from 2018 into mid-2021. We retained only nights with concurrent data for all three stations for this comparison, totalling 863 nights.

### (d) Modelling flight altitude and responses to atmospheric conditions

We used the thinned dataset to model the effect of atmospheric conditions and date on the volume and behaviour of birds across nights and altitudes using generalized additive models (GAMs) in the R package mgcv [47]. We constructed a single overarching model separately for each season and response variable (density and normalized density), with each thin plate regression smooth term allowed to interact with location. We used smooth terms for atmospheric predictor variables and date to account for nonlinear relationships with bird density. As model predictors, we considered: night of the year, height, tailwind, the U- and V- directional wind vector, air temperature, air temperature at ground level (taken from altitude bin closest to ground), specific humidity, turbulent kinetic energy and vertical air movement (omega). Including all locations in a single seasonal model allowed us to estimate the most parsimonious models and reduce model complexity when covariates have a similar effect at multiple radar stations.

We selected smooth terms using automated model selection with the dredge() function in the MuMIn R package [48] and removed variables that were strongly correlated. We removed from further candidate models the U- and V-directional wind vectors that were used to calculate wind speed and direction and our tailwind variable. We also removed air temperature because of its tight relationship with specific humidity and altitude, and turbulent kinetic energy because of its correlation with vertical air movement (Pearson correlation >0.7). We used a tweedie distribution with a log-link function and reduced the degrees of freedom of each smooth term by setting *k* = 6, and using restricted maximum likelihood estimation (REML).

For our models of nightly bird density across vertically integrated profiles of each night, we retained the predictors ‘night of the year’, ‘altitude’, ‘tailwind’, ‘air temperature at ground level’, ‘specific humidity’ and ‘vertical air movement’ plus a fixed term for the year and radar. We examined height-specific effects on flight behaviour by modelling normalized bird densities, defined as the altitude-specific bird density divided by the vertically integrated density (i.e. dens/*VID*). This normalization removes nightly variation in the numbers of birds, leaving only variation in altitudinal distribution, allowing us to examine the effects of each predictor variable on altitude selection only. These models were structured like those for nightly bird densities for vertically integrated profiles but using normalized density as the response variable and retaining only ‘height’, ‘tailwind’ and ‘omega’ as smooth terms after our model selection.

After selecting the predictor variables to use in our final models, we determined for each retained smooth term the evidence for geographical differences in responses at decreasing levels of spatial awareness: (i) differences between individual radar station, (ii) differences between Colombian stations versus a representative temperate radar in Illinois only, or (iii) no locational differences. We formally tested for presence of these levels of spatial difference for all variables by comparing between nested models that do and do not include an interaction term for locational differences with chi-squared tests in an ANOVA (electronic supplemental material). Specifically, we first tested whether a model with the variable grouped by all radar stations performed better than when grouped by all Colombia radars versus the Illinois radar or without any geographic grouping [49]. We completed this process separately for all four final models for each season and response variable, each containing different geographic groupings for the smooth terms (electronic supplementary material, tables S3 and S4).

We excluded the radar station in the Llanos region from our GAM analyses based on limited available data.

### (e) Validation with acoustic monitoring

As an independent measure of migration intensity, we recorded the vocalizations birds use to communicate during migratory flights (hereafter ‘flight calls’). We used an OldBird 21c microphone (Old Bird, Inc.) connected to a SWIFT automated recording unit (Cornell Lab of Ornithology) near the Magdalena Valley and Amazonia radars. We programmed the recorder to sample continuously throughout the night from 19:00 to 05:45 local time at a sample rate of 32 000 kHz, between 10 March and 15 May 2023 and between 22 August and 18 November 2021 in fall in the Magdalena Valley (on a residential roof in the city of Barrancabermeja) and from 25 February to 9 April 2022 for spring and 6 September–1 December 2022 for fall in the Amazon (on top of the Cerro Azul near San Jose del Guaviare). Our total recording effort amounted to 539 h across 30 nights during spring migration and 933 h across 64 nights during fall migration in the Magdalena Valley and 907 h across 51 nights during spring migration and 917 h across 58 nights during fall migration in the Amazon after removing periods with dead batteries or other technical problems.

We used the Nighthawk acoustic model to detect and classify flight calls in our recordings [37]. After using a custom R script to segment and manually review detections, we quantified the number flight calls over time while accounting for recording effort by dividing the number of calls per taxon in a recording by the number of hours in that recording for each night to calculate a nightly value of call rate. We then paired this acoustic measure of nightly migration intensity—call rate (calls per hour)—with radar-derived bird density, subsetting the radar dataset to nights when both the local radar and recording equipment were functioning concurrently (*n* = 297 nights). As with the radar data, we used GAMs to explore the relationship between calls per hour, radar-derived bird density and atmospheric conditions between locations and seasons. We generated two versions of these models: one group used call rate as the response variable and the other group used bird density as the response variable (electronic supplementary material, table S5). We transformed call rate to the fourth-root for modelling to improve normality of residuals. We used air temperature, tailwind, specific humidity, migration intensity (either call rate or bird density, depending on the model) and mean flight height as linear predictors, season and location as fixed effects and night of the year as a smooth term (electronic supplementary material, table S5). We were unable to directly compare radar and acoustic data during the spring 2023 season because processed radar data were unavailable.

## 3. Results

### (a) Nightly variation in migration traffic

**Figure 2.**
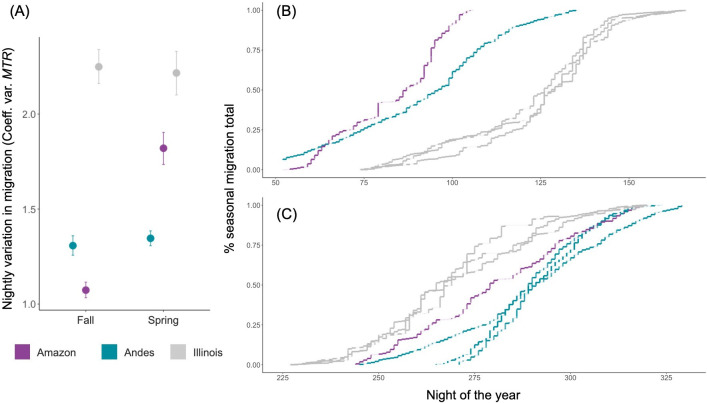
Low variation in nightly migration traffic drives a gradual accumulation of migration across a season in Colombia. (A) Comparison of coefficient of variation in nightly migration traffic rates between locations and seasons. Error bars show 95% confidence intervals and points are spaced along the *x*-axis to avoid overlap in plotted data. Cumulative migration traffic across a season during the boreal spring (B) and fall (C) in the Magdalena Valley (teal) and Amazonia (violet) in comparison with northern Illinois, USA (grey). Each line represents a time series for a different year, with each step in the time series corresponding to one night. The beginning and end of each line reflect the availability of data during that migration season. Periods with missing velocity data that prohibit calculating migration traffic rate are shaded as transparent.

Between-night variation in migration traffic was considerably lower in Colombian than temperate radars (figure 2A, electronic supplementary material, figure S4 and table S1). During spring migration, half of the total seasonal migration passage (averaged across years) occurred on 26% of nights in each season in the Magdalena Valley and on 16% of nights in the Amazon, in comparison with 9% of nights in Illinois, 12% of nights in New York and 8% of nights in Nebraska. During fall migration, half of the seasonal total occurred on 24% of nights in the Magdalena Valley and 25% of nights in the Amazon versus 9% of nights in Illinois, 12% of nights in New York and 6% of nights in Nebraska. That is, approximately twice as many nights were required to capture half the seasonal total of migration in Colombia as at representative temperate locations, except for spring migration over the Amazon, which was similarly episodic (electronic supplementary material, table S1).

We found that the timings of the seasonal peaks of migration in Colombia, defined by the dates by which 50% of total seasonal migration had occurred, were spaced approximately one week apart between our northern- and southern-most Colombian radar stations. That is, spring migration peaked earlier in the Amazon (mean peak on 28 March) and later in the Magdalena Valley (mean peak on 6 April). Similarly, fall migration peaked earliest in the Magdalena Valley (mean peak on 11 October) and latest in the Amazon (mean peak on 21 October).

### (b) Effects of weather on migration intensity and altitude selection

In our statistical models of nightly migration intensity, the only variable with a strong effect on bird density in both spring and fall across sites was date (electronic supplementary material, table S3). Tailwind, surface temperature, humidity and vertical air movement had significant effects on nightly bird density, but only in specific site–season combinations (electronic supplementary material, figures S5, S6 and table S3).

**Figure 3.**
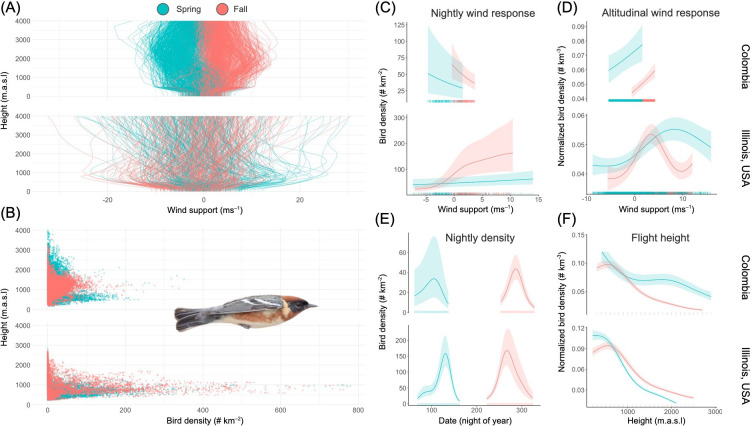
Migrating bird responses to wind regimes in tropical versus temperate environments. (A) Vertical profiles of wind support at the three focal radar stations in Colombia (top) and a radar in Illinois, USA, coloured by season. Tailwind is evaluated based on birds seasonally preferred migratory direction, following the EQ_airspeed_ equation in [45]. Note that winds are more consistent within spring and fall in Colombia compared to the considerable variation across altitude and season in Illinois. B) Mean flight altitudes of birds over Colombia (top) versus Illinois (bottom), colored by season. Each point in A and B corresponds to a single radar volume. C,D,F) Relationship between bird density and wind support, with rug plots indicating range of available conditions as predicted by GAMs modelling bird density in a single volume taken 3 h after sunset to capture effects across nights and C) bird density normalized across nights to capture effects across height only D,F). (E) Predicted nightly bird density across migratory seasons. (C–F) Top panels show data from the Magdalena Valley in Colombia and bottom panels show data from Illinois, USA. *Setophaga castanea* image courtesy of I. Davies.

We found that tailwind had a weak to moderate effect on nightly migration intensity in Colombia, with birds preferring to fly on nights with calm conditions regardless of whether the wind direction was supportive or opposing to their migratory direction (figure 3C). We found the strongest support for favouring calm conditions over the Amazon during boreal spring, where north-bound migrants consistently faced headwinds (figures 1C and 3A, electronic supplementary material, table S2). In the Magdalena Valley, where there was the least seasonal variation in wind direction, tailwind had a negative relationship with nightly bird densities. In contrast, wind had a positive effect on nightly bird densities over Illinois, USA, particularly in the fall, with a similar variation in wind direction available for birds to select from during both seasons (figure 3A,C).

While wind support generally had a moderate effect on the nights selected for migration in Colombia, it had a stronger effect on the heights selected for flight (figure 3F,D, electronic supplementary material, S6 and table S4). In the boreal spring, birds flew mostly below, but sometimes above, the layer of opposing trade winds (figure 3B). As a result, mean flight height was higher in the fall but more variable in spring (electronic supplementary material, table S2 and figure S9b). Birds also flew higher overall in Colombia than in Illinois, where densities decreased more strongly with altitude and were consistently below 2000 m (figure 3B,F). Birds more readily flew at high altitudes in the range of 3000−4000 m in Colombia (e.g. electronic supplementary material, figure S3).

Height and vertical air movement (omega) also had a significant effect on normalized bird density in our models of altitude selection (electronic supplementary material, table S4). Flight heights varied most between sites during spring but were similar during fall (electronic supplementary material, figure S7a and S7d). Birds modulated their flight altitude more relative to rising and falling air in Colombia than in Illinois during spring, but in the fall, birds consistently selected altitudes with rising air (electronic supplementary material, figure S7c and S7f).

Date was the best predictor of call rate in our acoustic dataset, outperforming weather variables in all our models (*p*-values < 0.001, electronic supplementary material, table S5 and figure S8). Call rates and bird density measured by the radar had a strong positive correlation (Pearson *R* = 0.46, *R*^2^ = 0.2, *p* < 0.001, electronic supplementary material, figure S8a).

### (c) Spatial variation in migration in Colombia

The magnitude, directionality and height of bird migration across Colombia varied by site and season (figure 1A, electronic supplementary material, figure S10). Both the total seasonal passage (i.e. migration traffic, electronic supplementary material, table S1) and seasonally averaged bird densities (electronic supplementary material, figure S9 and table S2) were higher in the inter-Andean Magdalena Valley and the Amazon than in the Llanos. Bird densities were higher during boreal spring than fall in the Amazon but were larger in fall than spring in the Magdalena Valley (figure 1A, electronic supplementary material, table S2). We also found significant differences in the densities of birds between years in the radar data (electronic supplementary material, table S3).

Bird ground-speed directions in Colombia generally corresponded with the northwest–southeast flow expected of birds travelling between Colombia and the rest of South America (figure 1A, electronic supplementary material, figure S10 and table S2). In the Magdalena Valley, birds predominantly flew a north–northeast trajectory in spring, which aligns with the direction of the Magdalena River. In the fall, birds flew more east towards the Cordillera Oriental of the Andes. Birds over the Amazon tracked deeper into the continent in the fall and northwest towards the Andes in spring. Birds also migrated predominantly northwest over the Llanos in spring and south in the fall.

We recorded substantially more flight calls in spring than fall at both recording stations, with spring means of 89 calls per hour in the Magdalena Valley and 75 calls per hour over Amazonia, contrasting with lower fall means of 18 calls per hour over the Magdalena Valley and 21 calls per hour over Amazonia (*p*-values <0.001).

## 4. Discussion

Scientists have long asked how reduced seasonality in climatic conditions shapes patterns of biodiversity at tropical latitudes, but the effects of tropical climates on animal migration are rarely considered [30,50–52]. We investigated how atmospheric conditions in Colombia affect the volume and phenology of bird migration for the first time in South America. We found that migration dynamics differed markedly from those at temperate latitudes: in Colombia, stable wind regimes affected altitude selection but had a relatively weak effect on the intensity of nightly migration, resulting in low nightly variation in migration traffic and a gradually changing migration intensity throughout the season in most cases (figures 2 and 3). However, birds took advantage of breaks in prevailing headwinds during spring over the Amazon, with an episodic phenology similar to temperate latitudes. We also found spatial variability in the volume and direction of migration, with the highest number of birds moving through the inter-Andean Magdalena Valley during the boreal fall (figure 1A). Our results highlight how stable regional climate shapes the dynamics of bird migration across a tropical landscape.

Wind assistance is an invaluable resource for migratory birds that must conserve energy on their long journeys. At temperate latitudes, highly variable winds mean that favourable conditions for migration occur on relatively few nights during a migration season, thus birds generally wait for profitable winds before departing on migration [3,19,53,54]. In contrast, the seasonally stable wind regime in Colombia means that birds have fewer conditions to select for and may not have to strategically time fueling and departure to match constantly changing local wind conditions. This difference is illustrated by our finding that more than twice as many nights are required to capture half of seasonal passage through our highest traffic site, the Magdalena Valley, than at three temperate radars in the USA and the vast majority of other radar stations in the continental USA, where half of migration occurs on 10% of nights in a migration season [19]. However, east of the Andes in the Amazon basin, the prevailing northeast to southwest winds are a consistent opposing force to birds migrating north in the boreal spring. We found that birds over the Amazon took advantage of lapses in these prevailing spring headwinds, leading to variable nightly migration traffic and a greater percentage of passage occurring on fewer nights within a season (electronic supplementary material, table S1 and figure 2). While this pattern of variable and episodic nightly migration is similar to what we found at temperate latitudes, it is caused by deviations from an otherwise stable wind regime in Colombia, rather by than a highly variable wind regime in temperate North America and Europe. Examining vertical profiles across a week of peak migration relative to wind support illuminates how birds modify their behaviour in this wind regime (electronic supplementary material, figure S11). Less variable nightly migration in Colombia may also be a result of the species composition of birds aloft. At temperate latitudes, shorter-distance migrants that overwinter in mid-latitudes may more readily adjust their migration strategies based on local weather than the longer-distance migrants featured in this study that cannot afford to wait for preferred conditions and are thought to have less plastic migratory behavior [55–57].

While the weak relationship between wind support and bird density between nights received moderate statistical support, we found a strong positive relationship between tailwind and bird density when controlling for nightly migration intensity, indicating a positive effect of supportive winds on flight altitude selection across Colombia (figure 3D). In the absence of significant atmospheric structure or topography, birds have a demonstrated preference to fly closer to the ground, where it is easier to track geographic features, respond to inclement weather or predation and regulate water loss, but they will ascend to higher altitudes to navigate mountainous regions and exploit supportive winds [22]. Both of these conditions are met in Colombia, where birds must cross or circumnavigate the Andes and the Orinoco Low Level Jet (OLLJ) creates a consistent layer of south-westward wind centred around 1500−2000 m.a.s.l. at night [23] (figure 3A). Similar to studies in the trade wind zone [21,22,58–60], birds leveraged this layer of tailwinds by flying higher on their southward migration in the boreal fall, and flying mostly below, but sometimes above the layer during boreal spring (figure 3, electronic supplementary material, figures S2, S4 and table S2). The seasonal height difference associated with this low-level jet likely underlies the higher volume of flight calls that we detected during the boreal spring in both the Amazon and the Magdalena Valley (electronic supplementary material, figure S8). Our analysis also indicates fall bird densities decreasing with height, albeit at a slower rate than in the spring, rather than peaking at the altitudes with the most supportive winds (figure 3F). This suggests that birds migrating over Colombia balance migrating close to ground level with selecting optimal altitudes for wind support, flying only as high as necessary to reach acceptable conditions above opposing winds—a trade-off also seen at temperate latitudes [17,18]. Typical surface winds at our focal sites in Colombia were generally calm, but when opposing winds strengthened at low altitudes we saw birds ascend to altitudes well above 2500 m.a.s.l. (e.g. electronic supplementary material, figure S3).

Birds’ decisions about when and where to fly are particularly complex in mountainous regions such as Colombia owing to the direct effects of topography and associated secondary effects of weather. We found the highest volume of migration in the Magdalena Valley during fall migration (figure 1A), one of two major inter-Andean valleys that run south to north through Colombia. These valleys act as natural funnels that increase bird densities between potentially volatile mountain ranges [61]. Furthermore, the Colombian Andes are a destination for the largest diversity of boreal migrants in South America [62], which also helps to explain the higher volume of migration over this station, and that in fall the dominant flight direction was directly towards the slopes of the eastern Andes (figure 1A and electronic supplementary material, figure S9). However, local topography affects wind and precipitation patterns at a fine spatial grain that may determine which key mountain passes and river valleys are available to birds, driving seasonal and annual differences in migration intensity that are not captured by our dataset. For example, tailwinds may be more important for nightly migration decisions in the Andes than we find here, but in response to local conditions that are not captured by spatially coarse weather models. Clarifying the compounded effects of microscale and synoptic weather on migratory strategies is imperative for future research in the tropics, particularly in mountainous regions [5].

The flow of migration observed in our data informs broader flyway structure in South America and its geographic context. Previous work has shown a dominant pattern of entering South America through Central America during fall migration and both Central America and the western Caribbean during spring, such that birds typically enter and leave South America through a relatively narrow corridor between the Darien Gap and the Sierra Nevada de Santa Marta [25,63–68]. More birds arriving in northwestern Colombia in the fall may explain the higher volume of birds that we detected southbound through the Magdalena Valley in fall than in spring, and the lower number of birds in Amazonia in the fall than in the spring. Additionally, the dominant north–northeast track that we found in the Magdalena Valley in spring suggests that birds over this radar favoured the Caribbean coast over the Darien Gap as a departure point from South America. East of the Andes in the Amazon and the Llanos, where there are no large mountains and valleys to shape flight paths and where major rivers run west-to-east instead of south-to-north, birds adhered to generally north-westward directions in spring and south-eastward directions in fall (figure 1A). These directions from the eastern lowlands capture the expected pattern of birds moving deeper into South America in the fall but offer limited insight into where birds aim to depart the continent in the spring, inviting further study within Colombia and South America.

Capacity for monitoring biodiversity and aeroecological research with weather radars in South America is only increasing. The IDEAM, which operates the radars that generated the data for this study, recently moved their database to open access on Amazon Web Services, resolving previous constraints on storing data, maintaining complete time series and increasing the number of stations with available data [40]. The data available there conformed partly to the quality and curation standards recommended for biodiversity monitoring [41], however this could be greatly improved by continuous radar operation with consistent settings and data curation. In particular, the proportion of pixels without a radial velocity estimate was relatively high for biological scatterers, likely owing to excessively stringent quality control by the radar processor. Navigating gaps in our time series, shifts in radar settings and frequent absence or poor quality of velocity data were some of the primary barriers we faced in this study, reducing the power of our analyses despite abundant data. The second major challenge we faced was separating migratory bird signals from insects. Unlike in North America and Europe, biological signals in the nocturnal radar data from Colombia were frequently dominated by insects based on the pattern of low airspeeds, movement largely in conjunction with the wind and signatures in velocity imagery [69]. The magnitude of this strong insect component was frequently similar to or larger than bird signals, particularly given the lower volume of migratory birds aloft in Colombia relative to North America [30]. Our approach to separating bird and insect signals relies on accurate altitudinal wind information and reasonably accurate assumptions on birds’ and insects’ self-propelled airspeed [14]. Greater coverage by weather balloons and other open-source repositories of vertical profiles of wind and other atmospheric data in Colombia and throughout South America would significantly enhance the potential for accurately classifying volant animal taxa in Neotropical weather radar products [41]. Similarly, increased acoustic monitoring for nocturnal flight calls would reveal which species are aloft, informing the airspeed values selected by scientists for this classification method.

Despite these challenges, our study is the first to quantify the flow of bird migration across South America using weather surveillance radars and acoustic monitoring. By showing a reduced effect of wind assistance and a strong effect of season and locality on where and when birds are traversing Colombia, we highlight the steady pace of migration in a tropical climate at a regional level. However, better understanding how the regional flow of migration is affected by microscale weather induced by topography is imperative to developing actions that lower risks for migratory birds across their annual cycle as they navigate a human-altered world [7,30]. The high density of both cities and migratory birds in Colombia means that migrants are more likely to traverse an urban environment in Colombia than in any other South American country [70]. However, a reduced relationship with forecastable weather conditions in Colombia makes coordinated mitigation to threats such as building collisions in cities challenging, particularly in the Andes where climate and topography are exceptionally complex. Increasing the spatial resolution of avian radar products and incorporating fine-scale patterns in precipitation can potentially improve our ability to observe and predict important high bird density fall-outs [71]. Continuing to leverage weather surveillance radars in Colombia and elsewhere in the tropics is imperative to addressing urgent conservation needs and understanding the aeroecology of earth’s most biodiverse latitudes.

## Data Availability

The code and data used in our analyses may be found in a Dryad repository [72]. Supplementary material is available online [73].
